# Rehabilitation Exergames: Use of Motion Sensing and Machine Learning to Quantify Exercise Performance in Healthy Volunteers

**DOI:** 10.2196/17289

**Published:** 2020-08-18

**Authors:** Reza Haghighi Osgouei, David Soulsby, Fernando Bello

**Affiliations:** 1 Imperial College Centre for Engagement and Simulation Science (ICCESS) Faculty of Medicine, Department of Surgery and Cancer Imperial College London London United Kingdom; 2 Children's Services and Dietetics Chelsea and Westminster Hospital London United Kingdom

**Keywords:** rehabilitation exergames, performance assessment, similarity score, motion sensing, machine learning, dynamic time warping, hidden Markov model

## Abstract

**Background:**

Performing physiotherapy exercises in front of a physiotherapist yields qualitative assessment notes and immediate feedback. However, practicing the exercises at home lacks feedback on how well patients are performing the prescribed tasks. The absence of proper feedback might result in patients performing the exercises incorrectly, which could worsen their condition. We present an approach to generate performance scores to enable tracking the progress by both the patient at home and the physiotherapist in the clinic.

**Objective:**

This study aims to propose the use of 2 machine learning algorithms, dynamic time warping (DTW) and hidden Markov model (HMM), to quantitatively assess the patient’s performance with respect to a reference.

**Methods:**

Movement data were recorded using a motion sensor (Kinect V2), capable of detecting 25 joints in the human skeleton model, and were compared with those of a reference. A total of 16 participants were recruited to perform 4 different exercises: shoulder abduction, hip abduction, lunge, and sit-to-stand exercises. Their performance was compared with that of a physiotherapist as a reference.

**Results:**

Both algorithms showed a similar trend in assessing participant performance. However, their sensitivity levels were different. Although DTW was more sensitive to small changes, HMM captured a general view of the performance, being less sensitive to the details.

**Conclusions:**

The chosen algorithms demonstrated their capacity to objectively assess the performance of physical therapy. HMM may be more suitable in the early stages of a physiotherapy program to capture and report general performance, whereas DTW could be used later to focus on the details. The scores enable the patient to monitor their daily performance. They can also be reported back to the physiotherapist to track and assess patient progress, provide feedback, and adjust the exercise program if needed.

## Introduction

### Background

Rehabilitation is essential to regain lost or weakened functionality after injury or surgery. Although it is commonly initiated in a clinic and supervised by a physiotherapist, the prescribed therapeutic exercises will normally need to be practiced at home by patients on their own. Lack of motivation and compliance may hinder the healing process and, in some cases, even worsen the injury. Advances in virtual reality technologies have resulted in various virtual rehabilitation platforms introduced to address this issue [1-3]. They are generally equipped with sensory devices to track and monitor the patient’s movement when performing the exercises. Among them are systems based on the concept of *exergaming* and exercise gaming. These are interactive video games with some simple scenarios that enable a patient to perform a therapeutic exercise by playing the game. Exergames are a subgenre of serious games developed for the purpose of encouraging exercise and activity. Although sharing some aspects with medical simulation, in exergames, the emphasis is more on the added educational value of fun and entertainment. Accessibility (not needing the constant presence of a physician) and entertainability (turning repetitive tasks to playful activities) are 2 key advantages of such systems.

The impact of serious games on physical therapy has been studied in terms of effectiveness [4-6] and motivational determinants [7,8]. However, as a virtual guide replacing or complementing a real physician, exergaming systems tend to lack objective, clinically meaningful evaluation of patient performance. At best, game scores, the extent to which the player achieved the goals of the game, are reported at the end of the session along with statistics such as completion time. The work presented in this study aims to fill this gap by introducing an approach to compare a patient's performance with that of a reference, using the Medical Interactive Recovery Assistant (MIRA) rehabilitation platform. We explore 2 different machine learning techniques: dynamic time warping (DTW) and hidden Markov model (HMM). They have been widely used for gesture recognition to study acquired motion trajectories [9-14]. The former belongs to model-less time domain methods, whereas the latter is a model-based probabilistic technique for time-series analysis. Comparing the patient’s trajectory with that of a physiotherapist as a reference, each approach generates an objective similarity score indicating how similar the performance was.

### Related Work

#### Rehabilitation Platforms

The concept of exergaming enables exercising when playing games. For players, it is an opportunity to play games in a more active and less passive manner. For patients, it offers the opportunity to practice therapeutic tasks in a more playful and less repetitive manner. Exergames offer various activities, such as aerobic exercises and dancing; balance and stretching workouts; and recreational simulations, such as golf, skiing, and more. However, they require additional hardware and software. In terms of hardware, they require proper sensory equipment to track the user's motion. In terms of software, the game scenario must accommodate whole body interaction. There are various commercially available game consoles that enable exergames, including Xbox (Microsoft), PlayStation (Sony), and Wii (Nintendo). Each comes with its own dedicated input device for enabling user interaction with the games, that is, Kinect for Xbox, Move for PlayStation, and Remote Plus for Wii.

Among them, Kinect has gained higher popularity owing to its acceptable performance and versatility [15]. Microsoft introduced Kinect in 2010 as a peripheral input device for its gaming console Xbox and discontinued it in 2017. Kinect enables interaction with virtual environments using gestures rather than conventional controllers. The device includes an RGB camera and a depth sensor, which combines full body 3-dimenonal (3D) motion capture capabilities and gesture recognition. Using the Kinect SDK 2.0, the position of 25 human skeleton joints can be accessed with a sample rate of 30 fps. Since its launch, researchers have used the Kinect for various applications, including rehabilitation [16-21]. Other similar motion-sensing devices capable of tracking the 3D position of the joints include Azure Kinect (Microsoft), Astra (Orbbec), RealSense (Intel), Structure Sensor (Occipital), and BlasterX Senz3D (Creative).

Kinerehab was introduced by Chang et al [16] to assist therapists in rehabilitating students at a public school. By conducting a user study with two patients, the authors compared two experimental phases, baseline (without assistive technology) and intervention (with Kinerehab), lifting both arms to the front, to the side, and upward. The data showed a significant increase in motivation (willingness to keep practicing) among participants and hence improved exercise performance using Kinerehab compared with performance in the presence of a therapist. A Kinect-based serious game for physiotherapy (KSGphysio) was proposed by Duarte et al [17] that had a mobile interface to facilitate the analysis of patient progress by generating relevant statistics. Cary et al [18] developed a web-based serious game called Therasoup to improve the patient’s motivation when performing exercises and to provide technical data to the physiotherapist, which helped in the assessment. The game tries to simulate daily life activities such as cooking, where the player controls an avatar to pick the ingredients from shelves and put them in a pan at the center. Su et al [19] developed a Kinect-enabled home-based rehabilitation system (KEHR) to assist patients in conducting safe and effective off-hospital rehabilitation without the immediate supervision of a physician. KEHR supported 3 different shoulder rehabilitation exercises: shoulder abduction, shoulder anterior elevation, and shoulder external and internal rotation. A serious game framework for therapy (Theragame) providing options to imitate the actions performed by an avatar or to play a game that trains specific parts of the body was introduced by Ferreira et al [20]. A web-based platform for physical telerehabilitation for patients after hip replacement surgery was described by Rybarczyk et al [21] with 2 goals in mind: making use of a low-cost motion capture device (Kinect) and real-time automatic assessment of performance. A comprehensive review of the technical and clinical impacts of the Kinect in physical therapy and rehabilitation is given by Mousavi et al [15]. The survey covers rehabilitation systems before and after Kinect as well as platforms with and without clinical evaluation. Although Kinect-based rehabilitation systems are accepted by both patients and therapists, the lack of objective, clinically meaningful evaluation of performance raises questions regarding their effectiveness.

Commercially available rehabilitation platforms based on Kinect and exergames include MIRA [1], VirtualRehab [2], and REHABILITY [3]. All these platforms have the capacity to be used at home to encourage and monitor performance or at a hospital to assist and support physiotherapists. MIRA is a class I medical device that uses games built based on best clinical practice and expertise from specialist physiotherapists to keep patients engaged and motivated throughout the therapy. VirtualRehab, a Conformitè Europëenne–certified class I medical device, is a product that can be used in clinics and hospitals as well as in patients’ homes, allowing them to continue their rehabilitation treatment. With REHABILITY, patients can perform rehabilitation exercises at the clinic and hospital or remotely, but with constant medical supervision. None of these systems offers automatic, objective assessment comparing patient performance with that of a reference. They offer game scores such as number of hits and statistics such as execution/completion time.

#### Performance Evaluation

Automatic performance evaluation of a user carrying out a task has always been a challenge among researchers in both medical and nonmedical domains. Such evaluations are usually subjective and, in the real world, are performed by judges who are experts in the given field. For example, evaluation of the quality of a dance, a gymnastic performance, or a physiotherapy/rehabilitation exercise may be performed by expert dancers, sport athletes, and professional therapists, respectively. Such evaluations require the presence of human specialists who may not be easily accessible or affordable. In addition, the fact that the assessment is subjective indicates that a different expert might have a different opinion. The ability to conduct objective automatic evaluations that are repeatable is thus highly desirable. A real-world example is a video game called Just Dance, which is developed by the French company Ubisoft for Microsoft Xbox. Using the Kinect sensor, the players must mimic the onscreen dancer's choreography to a chosen song. The system then continuously evaluates in real time the quality of a user's dance movements in terms of being “Ok,” “Good,” “Super,” or “Perfect” and reports a total numeric score at the end [22].

Studies in the literature concerned with automated evaluation of therapy motions are scarce [23-26], and not much attention has been paid to the development of metrics for performance evaluation [27]. As a common scheme, a reference model is first captured as the ground truth. Then, a user's performance can be compared with the reference using machine learning approaches. A comprehensive taxonomy of the metrics for evaluation of patient performance in physical therapy was proposed by Vakanski et al [27]. The metrics are classified into quantitative and qualitative categories. Further, quantitative metrics are divided into model less (based on raw measurements of motions) and model based (based on a mathematical model of the motions). Of the reviewed metrics, root mean square distance, Kullback Leibler divergence, log likelihood, and Fugl-Meyer assessment were used to classify a set of 5 human motions captured with a Kinect sensor.

Using KEHR, Su et al [19] applied DTW and fuzzy logic to detect real time subjective discrepancies between the model exercise and the performance of the patient. Before applying either algorithm, the user's execution of the prescribed exercise was recorded under the supervision of a professional. Then, 2 factors were included for the assessment: (1) trajectory disparity, the motional path created by each joint over time, and (2) speed variation, the time used to complete a designated exercise. Applying HMM and defining an accept/reject interval, a method to detect deviations from normal repetitions in therapeutic activities was presented by Palma et al [23]. The authors later compared the performance of their HMM-based technique with that of DTW [28]. A similar approach using HMMs to assess the correctness of telerehabilitation exercises was employed by Deters et al [24], whereas a cloud-based physical therapy monitoring and guidance system that applies DTW to produce subjective assessments in terms of being too slow/fast or overdone/incomplete was proposed by Wei et al [25]. Richter et al [26] presented an error classification algorithm for therapy exercises based on incremental DTW to classify the incorrect motions in a hip abduction exercise into 4 discrete categories: bent knee, foot outside, upper body, and wrong plane. A variance of DTW called multi-template, multi-match DTW was used by Yurtman and Barshan [29] to detect and evaluate physical therapy exercises using wearable motion sensors, providing a quantitative measure of similarity between an exercise execution and previously recorded templates. A continuous time warping algorithm based on automatic motion assessment learning was introduced by Tal and Shimshoni [30]. The resulting models produced numerical scores comparable with those of the Fugl-Meyer assessment. Recently, a DTW-based algorithm was developed for assessing Kinect-enabled home-based rehabilitation exercises to support auto coaching in a virtual gaming environment [31]. Using a simple but innovative method, the DTW distances are converted to meaningful performance scores in terms of percentage. By conducting a user study, the scores are then validated with the expert ratings showing a strong positive linear relationship. In another recent work, a deep learning–based framework for the assessment of rehabilitation exercises was proposed [32]. The framework consists of algorithms for dimensionality reduction, performance metrics (based on the Gaussian mixture model), scoring functions, and deep learning models. The authors demonstrated the capacity of the trained models by evaluating a data set of 10 rehabilitation exercises.

Similar techniques have also been employed in other domains, such as dance motion evaluation. Jang et al [33] employed DTW and Laban movement analysis to evaluate the correctness of dance movements in terms of being best, good, bad, and worst. In another work [34], the authors employed HMM for dance gesture recognition and evaluation, comparing the results with those of domain experts in terms of being good, medium, or bad.

A common factor among all these efforts was that they focused on evaluating the incorrectness of the performance on the basis of subjective terms. In most cases, the method developed was used to sort multiple erratic performances with respect to a reference template. This approach motivated us to explore the use of DTW and HMM to generate a similarity score between a participant’s performance and a reference.

## Methods

### Materials

#### MIRA

MIRA is a software platform that turns physiotherapy exercises into clinical exergames [35]. It aims to increase engagement levels and improve the uptake of exercises by converting the rehabilitation sessions into entertaining activities, making therapy more convenient and easier to follow, and offering greater accessibility. In turn, this has the potential for shorter recovery times as well as supporting physiotherapists, reducing workload, and waiting times at clinics. MIRA has been used in several clinical studies [36-39].

**Figure 1:**
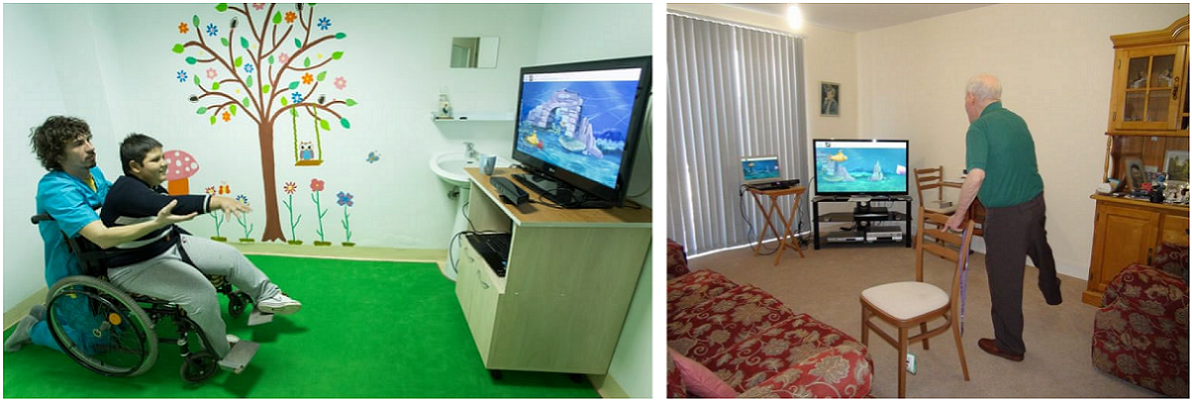
Medical Interactive Recovery Assistant system including a Kinect motion sensor and software to match an exercise with a game. A child (left) and an elderly gentleman (right) playing the Atlantis game. The child is practicing an arm exercise, the gentleman a hip exercise.

**Figure 2:**
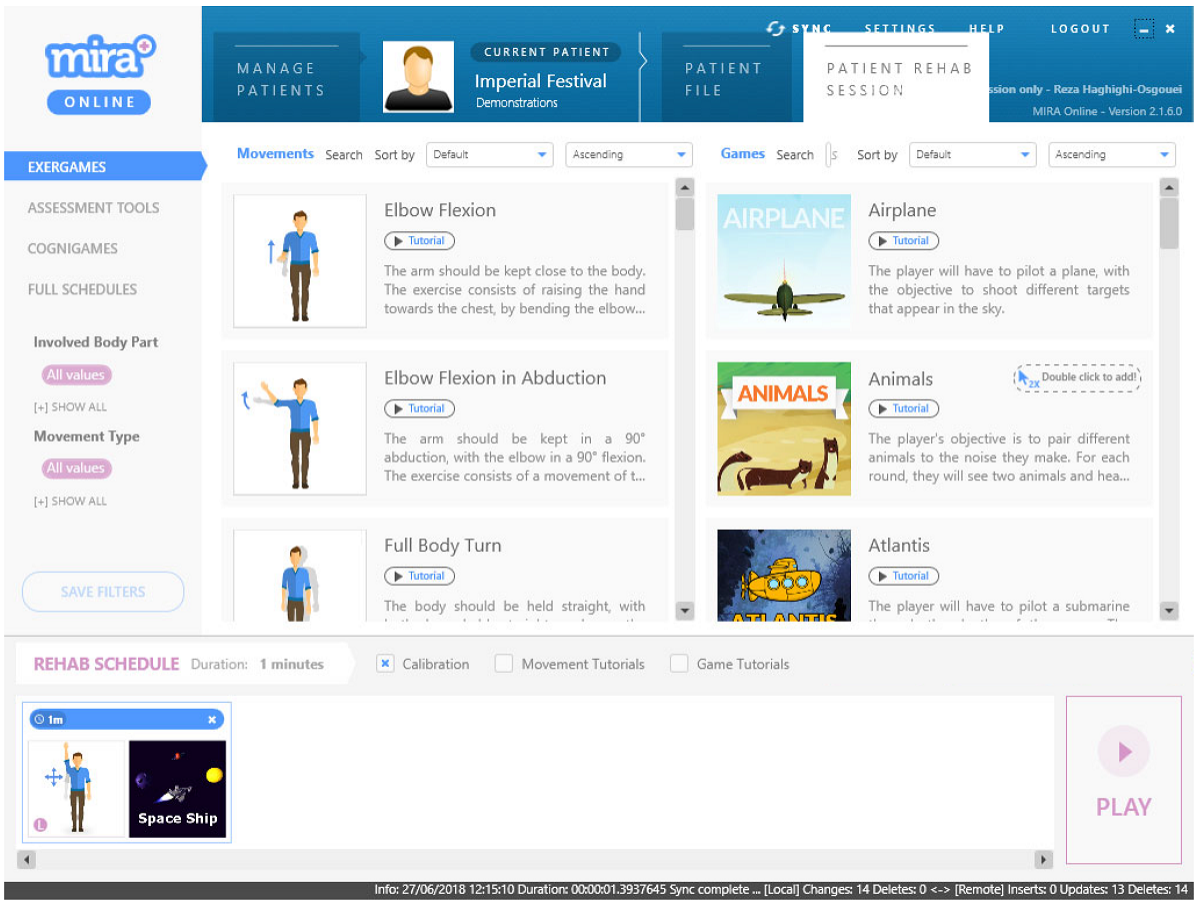
A snapshot of the Medical Interactive Recovery Assistant program. Exercises are listed on the left and games are shown on the right. Multiple game options allow practicing the same exercise with different games, thus encouraging patients to cope with the prescribed exercise by discovering the various game scenarios.

The MIRA system includes a Kinect V2 sensor (Microsoft Corp) connected to a computer running the MIRA program (Figure 1). Currently, 32 exercises and 25 games are supported by MIRA. Each rehabilitation session requires selecting an exercise and a suitable game (Figure 2). Exercises include shoulder abduction, elbow flexion, and side strides, and examples of games include Firefly, Fishing, and Football. Once an exercise has been selected, adequate game options are presented. The selected combination of exercise and game is then added to the session and can subsequently be executed. Each execution starts with a process of calibrating the patient’s position in front of the Kinect. A short video tutorial explaining the exercise is followed by another video tutorial describing the game mechanics. As the game starts, the user must play it by moving the intended body part (ie, left arm, right leg, or neck) in the manner shown in the video.

At the end of each session, the MIRA system reports various scores. Depending on the game, it reflects on the extent to which the player follows the game's objectives. For example, the number of fish caught and taken to the boat or the number of times the spaceship is safely passed through the fire rings. Although the scores can be an indication of how well the user played the game, they do not have much value in a clinical context. The aim of this work is to introduce an objective evaluation method that is more meaningful and suitable for clinical evaluation.

#### Data Collection

We developed a program in the Unity 3D game engine (Unity Technologies) [40] to capture and store raw 3D position coordinates of the selected joints using the Kinect. This was needed as the MIRA program does not allow accessing joint data when playing an exergame because of regulations imposed on class I medical devices. There were no technical problems executing both programs in parallel as the Kinect SDK permits simultaneous access to the sensor from multiple sources. Using Kinect V2, the 3D position coordinates of 25 different human skeleton joints can be tracked with an update rate of 30 fps. However, it is not necessary to track all the joints but only those that are involved in the chosen exercise. For this study, 4 different types of exercises were selected in consultation with a physiotherapist (DS): shoulder abduction (both left and right arms), hip abduction (both left and right legs), lunge (both left and right legs), and sit-to-stand exercises. This resulted in a total of 7 exercises to be performed by participants. A brief description of each exercise is given in Textbox 1.

**Figure 3:**
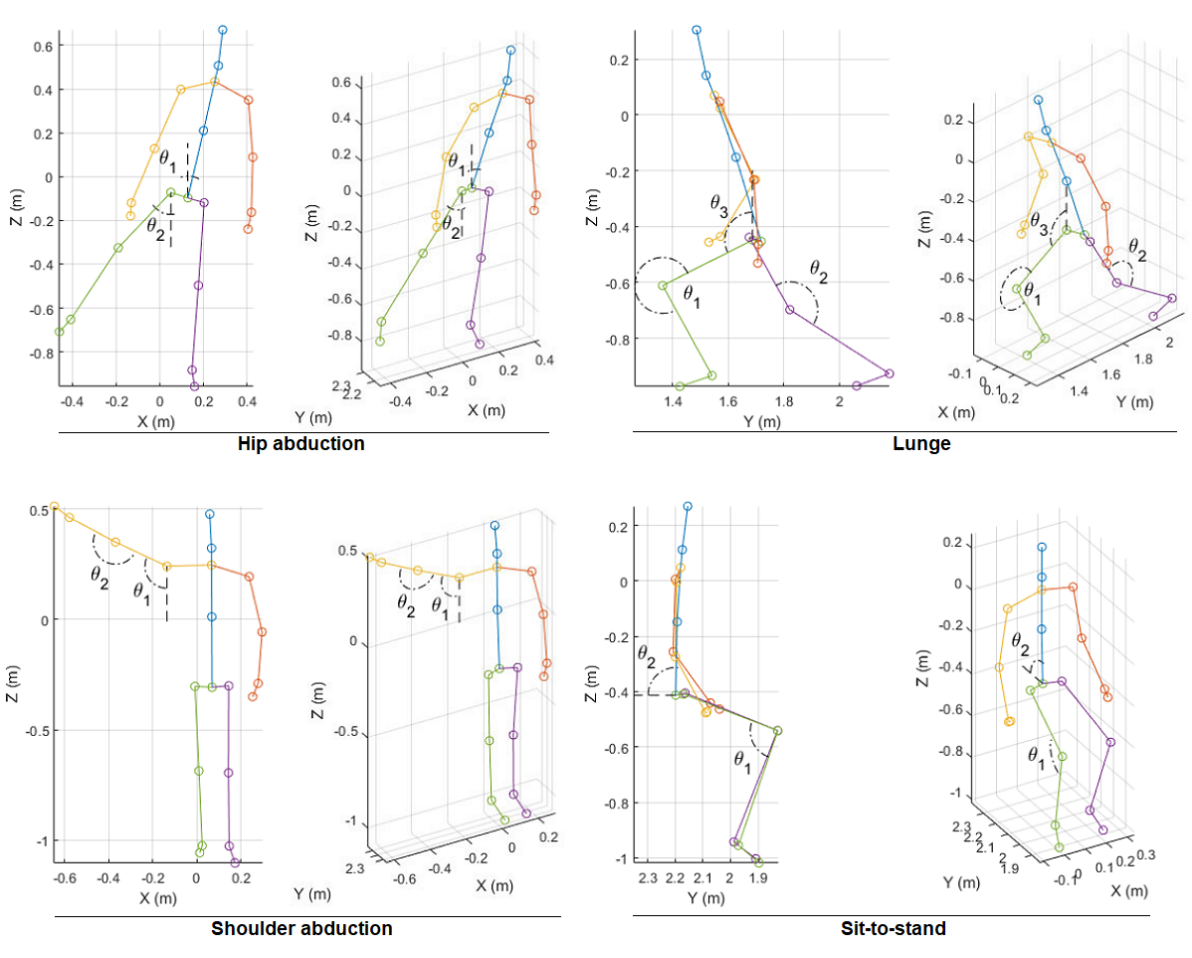
Extracted features (joint angles) from the joint 3D positions for each type of exercise. Both 2D side view and 3D perspective view are provided for clarity. 2D: 2-dimensional; 3D: 3-dimensional.

**Figure 4:**
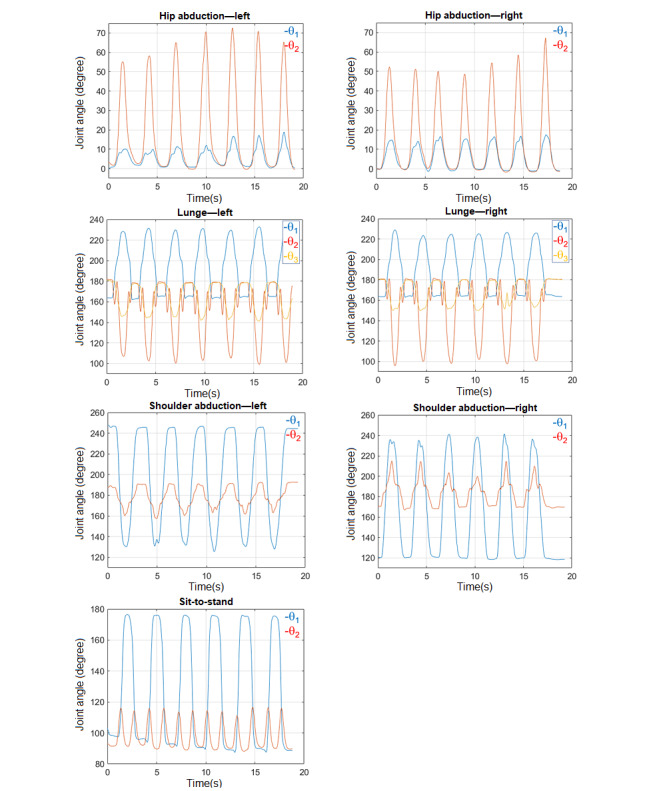
Sample extracted features obtained from 3D position for all seven exercises: hip abduction left (hpl) and right (hpr), lunge left (lul) and right (lur), shoulder abduction left (shl) and right (shr), and sit-to-stand (sit).

As 3D position coordinates are dependent on the user size and location in front of the Kinect camera, we decided to extract invariant features (joint angles) to describe each exercise optimally (Figure 3). These scalar features were discussed with the physiotherapist and confirmed to be sufficient for capturing the essence of each exercise. For example, in shoulder abduction, the shoulder angle (θ_1_) reflects the range of motion and the arm angle (θ_2_) indicates whether the arm is being stretched or not. Except the lunge exercise, which required 3 joint angles, all other exercises could be described with only 2 angles. A sample plot of the extracted features is shown in Figure 4.

A motion trajectory T(l) is formed by the sequence of feature values within the time frame 0≤t≤l, where l is the execution time. T(l) is a matrix of size l×3 for the lunge exercise and l×2 for rest. 


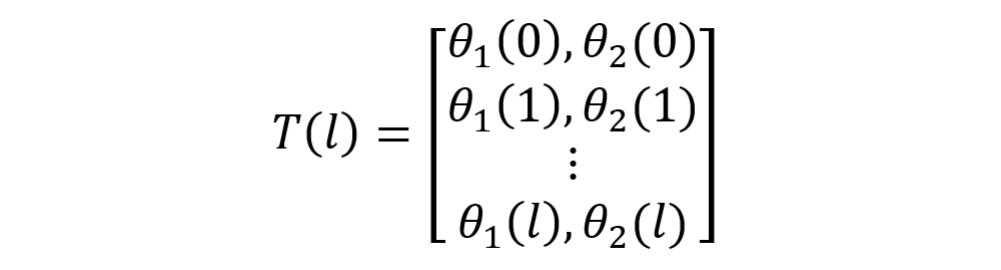

**Correct execution of each exercise.**

- Shoulder abduction: the arm should be kept close to the body. The exercise consists of raising the arm away from the side, keeping it in a straight line with the body.
- Hip abduction: the leg should be held straight and on the ground. The exercise involves raising the leg away from the side, keeping it in a straight line with the body.
- Lunge: stand straight facing forward with the spine and the pelvis in a neutral position. Take a step forward with a leg that is long enough so that when the knee bends, it does not go beyond the toes. Bend the back knee until it almost touches the floor, keeping both the torso and the spine in a neutral position. Return to the starting position.
- Sit-to-stand: sit on a chair. Without using the hands for support, stand up and sit back down. Make sure each movement is slow and controlled.

### DTW

DTW [41] is a technique that aligns 2 time series and determines the minimum Euclidean distance between them. It is a frequently used approach in speech recognition to classify sound waves of the same word spoken in different accents and durations. DTW is sensitive to both the signal pattern and the amplitude. If 2 signals have the same patterns, for example, the same number of peaks but different amplitude, then the alignment cannot be perfect, thus yielding a large distance between them. If they have the same amplitude but different patterns, the alignment will also result in a large distance. Therefore, the output distance is a measure of the similarity between 2 time series. The higher the distance, the greater the deviation.

Although DTW was initially applied to speech recognition, it has also been widely used in gesture recognition [10,12,42]. In these studies, the motion trajectories, each representing a gesture, are classified into the most similar gesture group (ie, the one with the smallest distance) by converting the distance between 2 trajectories into a similarity measure.

We define D_RP_=DTW(T_R_,T_P_) as a distance measure between the reference (T_R_) and the participant (T_P_) trajectories. The MATLAB function *dtw* was used for the implementation. Although the lower limit (D_l_) of this distance is 0 (being perfectly similar), the upper limit is unknown and can take any large value. By estimating an upper limit, it is possible to convert the distance measure into a similarity score. By associating the upper bound with the worst possible performance, an upper limit can be approximated. For all the exercises, not moving would be the worst performance. We refer to this worst trajectory as T_W_. Calculating D_RW_=DTW(T_R_,T_W_) allows us to establish the upper bound. Knowing both the lower and the upper bounds, a given distance D_l_=0≤D≤D_u_ can be transformed into a similarity measure or percentage score 0≤S_D_≤100:

S_D_=100×(D-D_l_)/(D_u_-D_l_)=100×(D/D_u_)

### HMM

**Figure 5:**
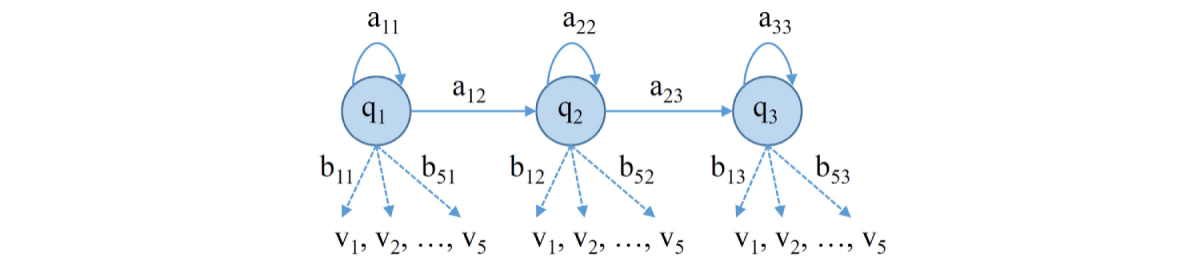
Hidden Markov model with three states (q1 to q3) and five observation symbols (v1 to v5). The relationship among states is described by transition matrix A=[aij]3x3, and between states and discreet symbols by observation matrix B=[bij]3x5. It is assumed the system evolves through certain states whose relationship is to be studied.

An HMM [43] is a stochastic model that considers an observed signal as the result of the transition of a system between several states, each of which has the probability that a particular symbol might be observed. HMMs are useful for the recognition of temporal patterns such as speech, handwriting, and gestures. An HMM with discrete observations is mainly specified by the state transition matrix A and the observation matrix B, assuming that the system goes through N different possible states S_1_, S_2_, ..., S_N_ and in each state, one of M different symbols v_1_, v_2_, ..., v_M_ can be observed (Figure 5).

For performance evaluation, a single HMM, λ_R_, is trained based on the reference motion trajectory T_R_. We then calculate the log likelihood of T_P_ given the trained model by L_RP_=log(P(T_p_|λ_R_))/l_p_. Similar to DTW, the lower and upper limits of the log likelihood need to be calculated. The upper limit (L_u_) is known and is equal to 0, as the highest probability is 1. However, the lower limit is unknown and can be any small value less than zero. Same as before, we assumed that this lower limit reflects the worst possible performance captured by T_W_. Letting the lower limit be L_RW_=log(P(T_w_ |λ_R_))/l_w_, the similarity score 0≤S_H_≤100 corresponding to log likelihood L_l_≤L≤L_u_=0 is obtained by:

S_H_=100×(L-L_l_)/(L_u_-L_l_)=100×(L_l_-L/L_l_

### Experimentation

#### Setup

Standard hardware (computer, television, Kinect sensor) was used in combination with a Unity program to display the participant’s live performance on the screen and store the 3D position data along with a time stamp. As mentioned above, 4 types of exercises were chosen to be performed by the participants: shoulder abduction, hip abduction, lunge, and sit-to-stand exercise. Except the sit-to-stand exercises, all other exercises were performed for both the left and the right sides, resulting in a total of 7 exercises.

#### Participants

A total of 16 healthy participants, including 8 adult females (22 to 30 years), 6 adult males (22 to 40 years), and 2 school boys (12 and 17 years), were recruited for the study. Participants were asked to stand in front of the Kinect sensor and perform each of the 7 exercises for 20 seconds. They were told to repeat the chosen exercise at least five times with a short pause between each repetition. In addition to the 16 participants, the physiotherapist involved in the project (DS) was asked to perform the exercises as the reference performance. He repeated each of the 7 exercises at least five times during a period of 20 seconds each.

## Results

**Figure 6:**
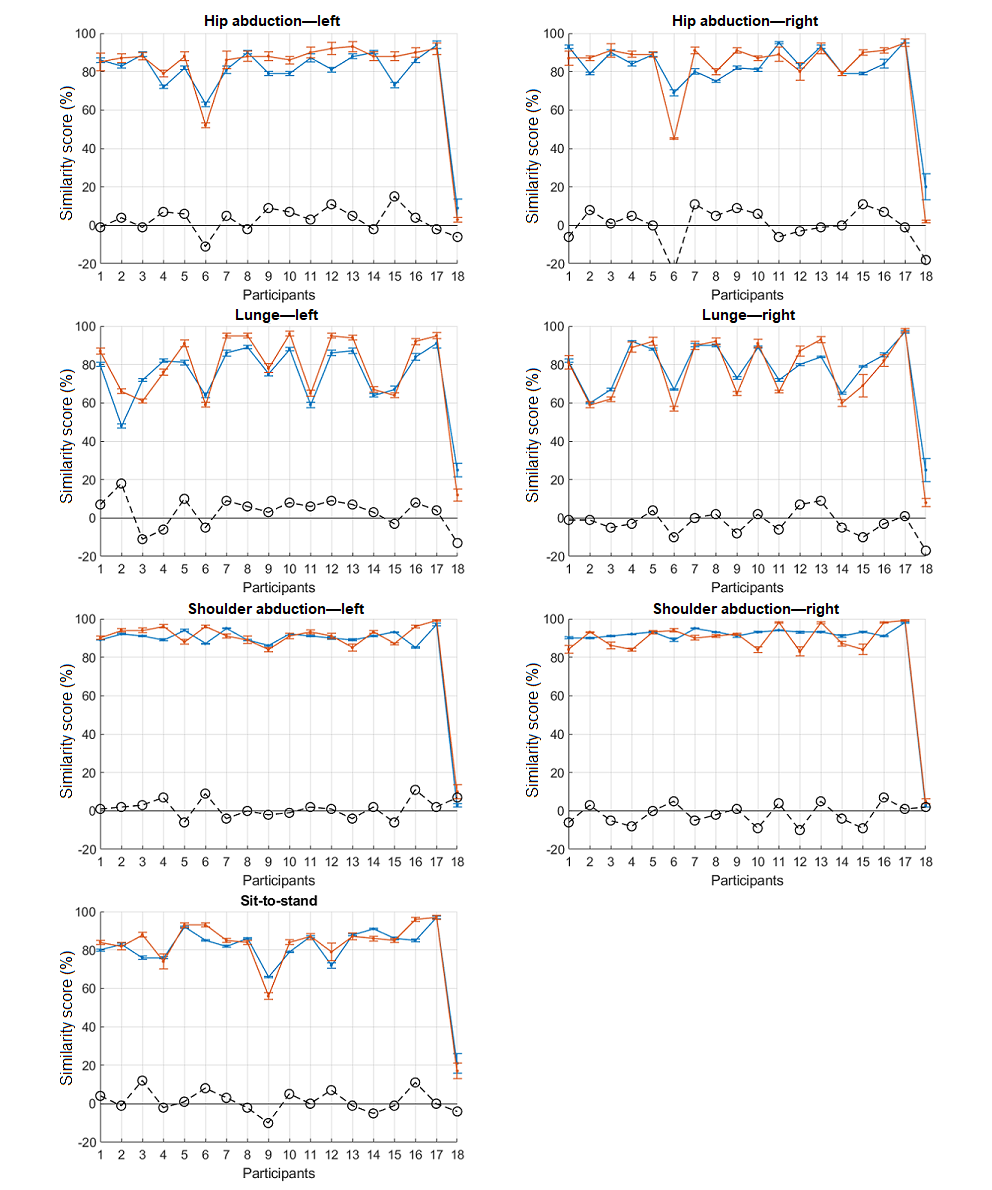
Similarity scores obtained from applying dynamic time warping (SD, solid blue line) and hidden Markov model (SH, solid red line). Their difference, SH-SD, is shown by the black dashed line. Participant 17 is the physiotherapist and 18 is the worst performance, that is, making no movement. Error bars indicate standard error.

Of the 5 repetitions, 3 were extracted (ignoring the first and the last) for each participant. For DTW scores, the distance between each repetition of a participant and each repetition of the physiotherapist was calculated, yielding a total of 9 values. The final DTW score (S_D_) was obtained by taking the average of these values. For HMM scores, the likelihood of each repetition of a participant given the physiotherapist’s model was calculated, yielding a total of 3 values. The final HMM score (S_H_) was obtained by taking the average of these values. Figure 6 shows the similarity scores obtained by applying DTW (S_D_, solid blue line) and HMM (S_H_, solid red line) for each exercise. The difference between the 2 scores, S_D_−S_H_, is also indicated in the plots by a dashed black line. Participant 17 is the physiotherapist and hence the reference. As expected, he obtained the maximum score in all cases. The worst performance, that is, making no movement, is included as participant 18, yielding very low scores in all exercises. Ideally, we would have expected to obtain a maximum score of 100% for the reference and a minimum score of 0% for the worst performance. However, these values were not obtained because of natural variability among the repetitions and cross-comparison between the repetitions.

## Discussion

Several observations can be made from these plots in Figure 6. Both scores showed similar trends. Whenever a participant did not perform well according to the DTW score (S_D_), his/her HMM score (S_H_) was low as well. Examples of this behavior are participant 6 in hip abduction (left and right) and participant 9 in sit-to-stand.

Regarding the difference, S_H_−S_D_, it is difficult to observe any obvious pattern. On some occasions, the difference was positive and on some others it was negative. There were also cases where the difference was negligible. The difference could be as large as +18% (participant 2, lunge—left) or as small as −24% (participant 6, hip abduction—right). For the reference participant 17, the difference was generally very low (−1%, −1%, 4%, 1%, 2%, 1%, and 0%). However, this was not the case for the worst performance, participant 18. In most exercises, the difference was negative and was not negligible, indicating that the S_D_ was usually larger than the S_H_ for the worst performance. In addition, in most plots, S_H_ was closer to 0% than S_D_ for the worst performance.

Among the exercises, shoulder abduction was less challenging and easy to perform, which is reflected by participants performing well and achieving high similarity scores. In contrast, lunge was the most difficult and demanding exercise to perform, which is also reflected in the obtained similarity values.

**Figure 7:**
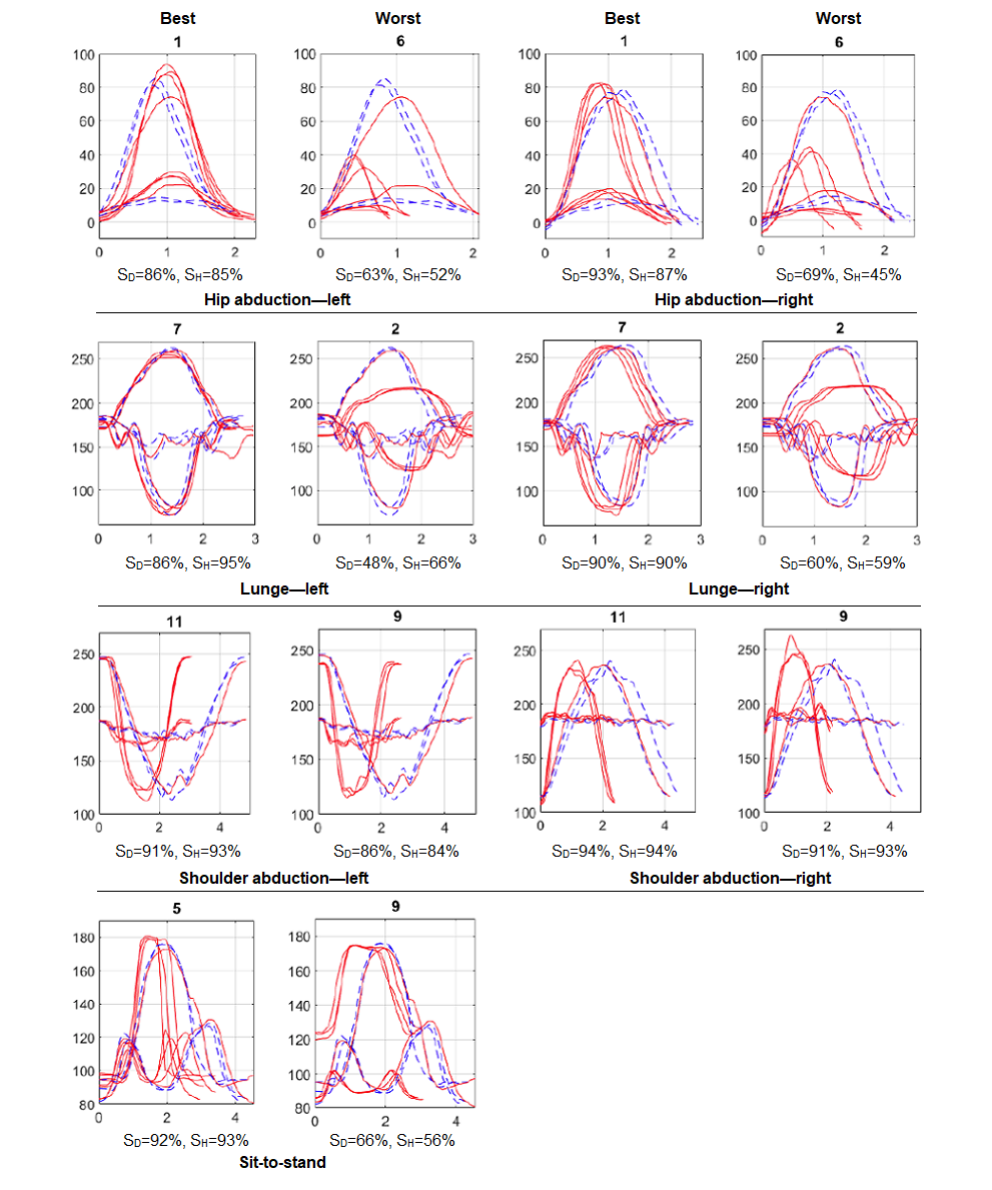
Time-domain plots of the best and worst trajectories (excluding participants 17 and 18). X-axis indicates the time in seconds and Y-axis indicates the joint angle in degree. The three repetitions of the reference trajectories are given in dashed blue lines and the chosen participant’s trajectories in solid red lines. All plots include two features, except for lunge where three features are presented.

Time domain plots of the best and worst performances can be used to visually examine the correlation between the trajectories and the calculated scores. For each of the 7 exercises, the trajectories of the best and the worst performances were plotted against the reference (Figure 7). Each plot illustrates 3 repetitions of the chosen participant (solid red lines) and 3 repetitions of the physiotherapist (dashed blue lines). It should be noted that for hip/shoulder abduction and sit-to-stand, each repetition includes 2 features, and for lunge, each repetition includes 3 features (Figure 4). Evidently, whenever the participant’s trajectories match those of the reference (in terms of both amplitude and duration), the obtained score is high. Conversely, when the patterns do not match, the score is low. For example, in hip abduction—left, participant 6 failed to achieve full range of motion (compare the peak amplitude, 40° versus 80°) and performed faster (compare the duration, 100 [1 second] versus 200 [2 seconds]). It is worth mentioning that both the DTW and the HMM algorithms are sensitive to amplitude than duration. They are both time-series algorithms that take into account the sequence of data (amplitudes) regardless of their frequency (duration). DTW does not allow time scaling of members within the sequence, and the HMM algorithm discards the time dependency of members by grouping them into clusters during the quantization preprocess. If two participants followed the required range of motion, but one performed slower (or faster) than the other, their score would be the same (see lunge—left, both participants 11 and 9).

**Figure 8:**
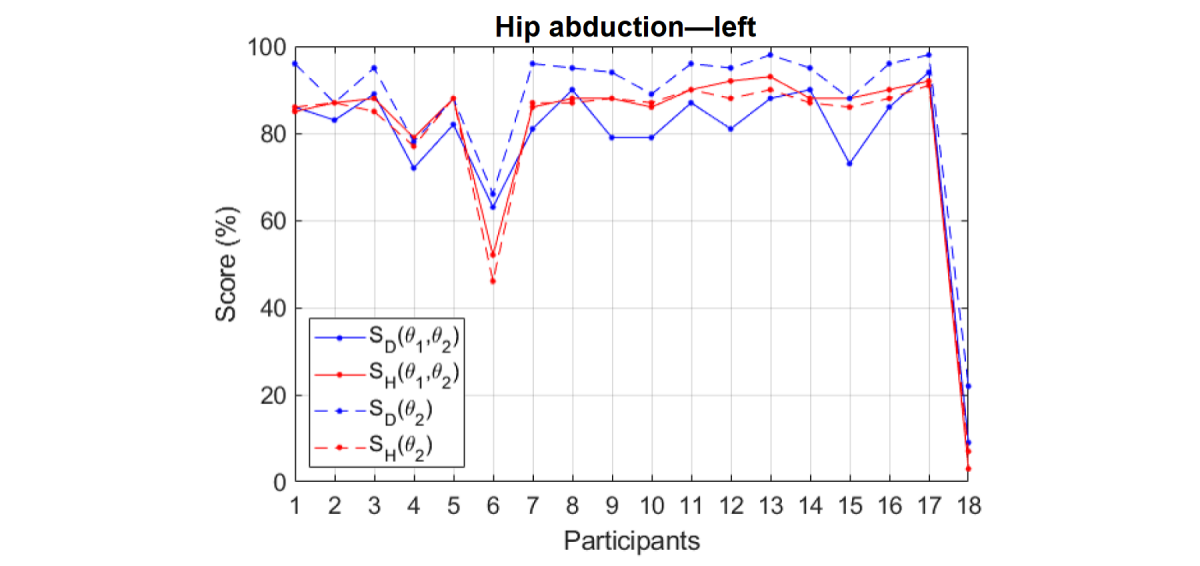
The effect on the scores of removing minor features.

Except the lunge exercise for which 3 features (joint angles) were extracted, 2 features were obtained for all the other exercises. However, not all the extracted features had the same weight and importance. For instance, in hip abduction, referring to Figures 4 and 7, θ_2_ (hip angle) is the more relevant feature and θ_1_ (torso angle) had lesser importance. This is contrary to the results for shoulder abduction and sit-to-stand. For lunge, θ_2_ and θ_1_ were both equally important, but θ_3_ was lower. The main feature has a higher influence on the obtained similarity score. This is shown in Figure 8 for hip abduction—left as an example. Generally, the same trend was maintained for each measure (compare the same color solid and dashed lines). However, the effect of removing a minor feature θ(_1_) was less influential on S_H_ than on S_D_, as DTW is more sensitive to detail than HMM.

Although single-feature S_D_ is clearly larger than full-feature S_D_, single-feature S_H_ is almost the same as full-feature S_H_. Adding more details, that is, presenting additional minor features, increases the distance values (and hence decreases the similarity scores) obtained by applying DTW.

**Figure 9:**
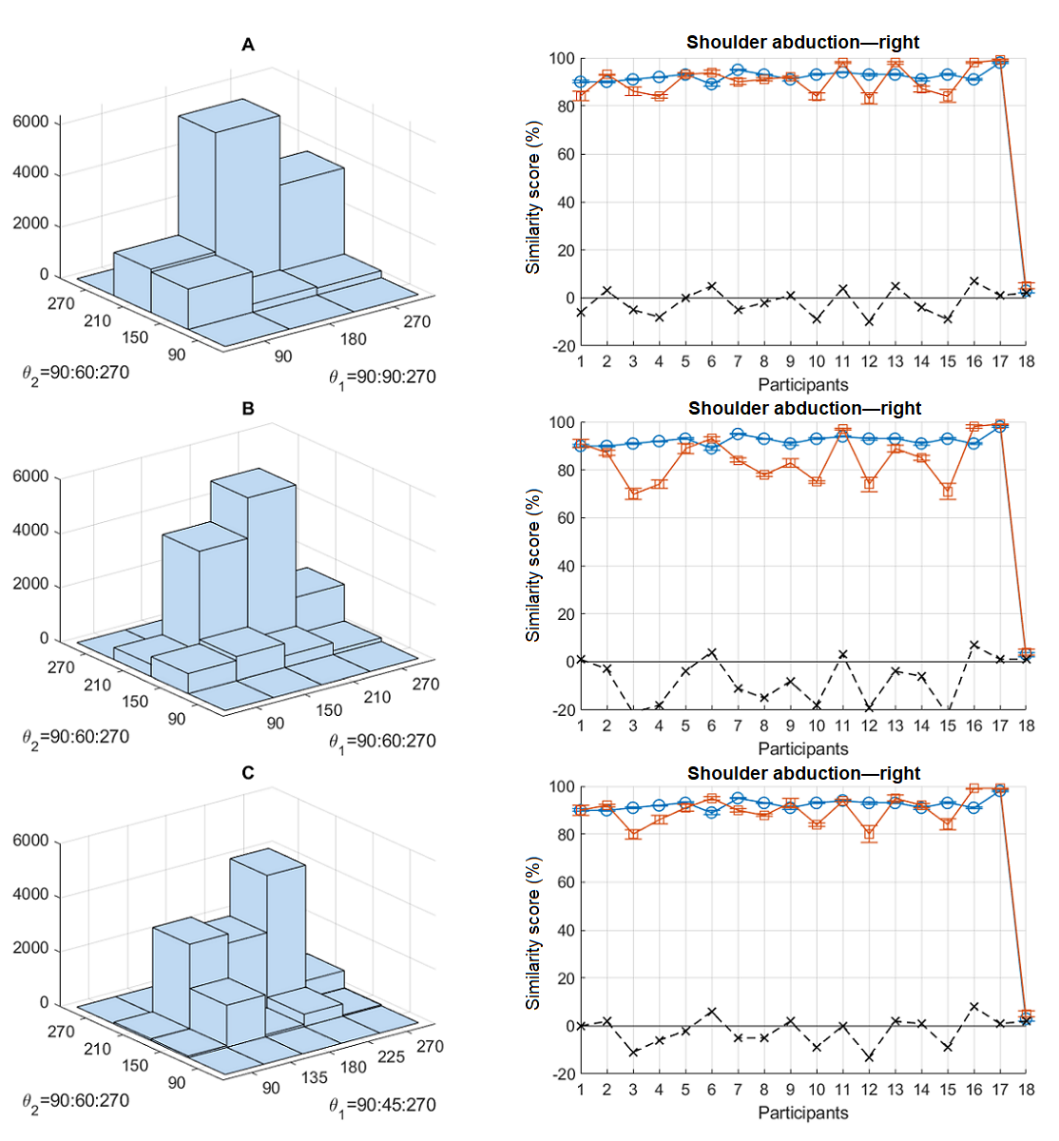
The effect of quantization on the calculated hidden Markov model scores.

Although DTW is more sensitive to detail, HMM is more sensitive to the way the feature space is quantized. Quantization is a preprocess applied over the extracted features to segment them into several clusters for the purpose of training a discrete HMM. The boundaries and the number of clusters have an obvious effect on the HMM scores. This can be seen in Figure 9 for shoulder abduction—right as an example. Bivariate histogram plots are used to visualize the clusters. Three cases were tested, changing θ_1_ (main feature) boundaries and keeping θ_2_ (minor feature) intact. θ_1_ is divided into 3 clusters (90°, 180°, and 270°) in case A, 4 clusters (90°, 150°, 210°, and 270°) in case B, and 5 clusters (90°, 135°, 180°, 225°, and 270°) in case C. In all cases, θ_2_ is divided into 4 clusters (90°, 150°, 210°, and 270°). A closer look reveals that the S_H_ scores in case B are clearly lower than those in cases A and C, which are more similar. The reason for this is that feature points are grouped into different clusters for the participant and the reference. This affects (reduces) the probability that a selected feature point of the participant is generated by the reference model. Most likely, the feature points of the participants and the reference in cases A and C are grouped into similar clusters; hence, their similarity scores are higher and more similar. In case B, however, they are grouped into different clusters, decreasing the probability values, thus lowering the similarity scores. One can take advantage of this behavior by adjusting the sensitivity of HMM similarity scores to smoothen or sharpen the differences.

**Figure 10:**
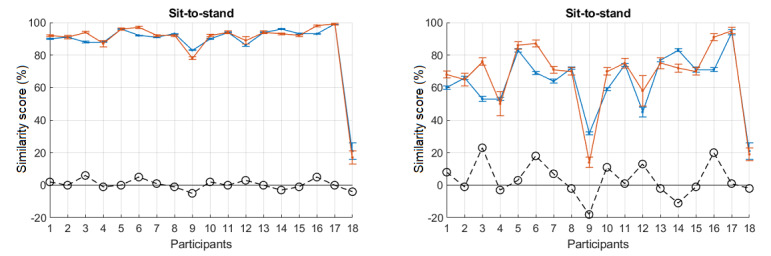
Effect of altering limits (Du and Ll, obtained from the worst performance) on the scores: multiplied by 2 (left) and divided by 2 (right). Error bars indicate standard error.

Worst performance is a key factor affecting the scores in both measures as it corresponds to the upper or lower boundary, as previously explained. This is evident from S_D_=100×(D-D_l_)/(D_u_-D_l_)=100×(D/D_u_) and S_H_=100×(L-L_l_)/ (L_u_-L_l_)=100×(L_l_-L/L_l_). Both unknown limits (D_u_ for DTW and L_l_ for HMM) are used as denominators to normalize the distance S_D_=100×(D-D_l_)/(D_u_-D_l_)=100×(D/D_u_) and likelihood S_H_= 100×(L-L_l_)/(L_u_-L_l_)=100×(L_l_-L/L_l_) values. The larger the denominator, the smaller the deviations (fluctuations) in the scores. This value can also be intentionally altered to adjust the sensitivity of the scores. With a larger denominator, the scores are smoother and the differences between participants become smaller. With a smaller denominator, the scores become sharper and the differences between participants are highlighted. As explained previously, we chose no movement as the worst performance for all exercises. Seemingly, a different worst performance can yield different scores if it generates different denominators. For example, one might say that closing the elbow in shoulder abduction could be worse than keeping it stretched (the current situation). An example of altering limits (multiplying and dividing D_u_ and L_l_ by 2) for sit-to-stand is shown in Figure 10. When the limits are magnified (multiplied by 2), the scores are increased and smoothed (Figure 10, left). When the limits are shrunk (divided by 2), the scores are decreased and sharpened (Figure 10, right). Smoothed scores can be used to encourage patients performing prescribed exercises in the early stages, whereas sharpened scores could be used in later stages to encourage further mastering of the skills involved in performing the exercises.

**Figure 11:**
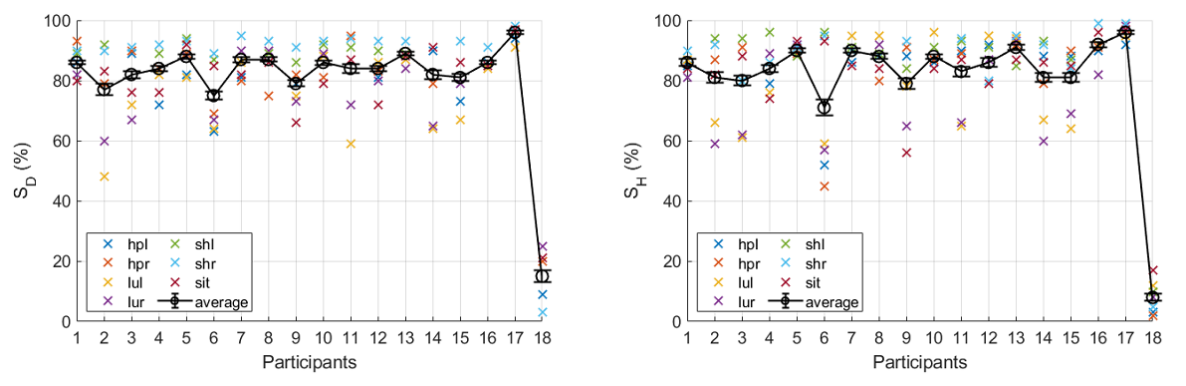
The combined and averaged scores (left: SD and right: SH) for all seven exercises. Error bars indicate the standard error.

Several comparative tests were also conducted. Figure 11 shows the average scores across all 7 exercises. For each participant, the average score was obtained from 9×7=72 scores (9 values for each of the 7 exercises). As can be seen, the 2 plots show very similar trends. This was verified by applying the *t* test over the 2 measures (*P*=.49). Small error bars indicate high levels of consistency.

**Figure 12:**
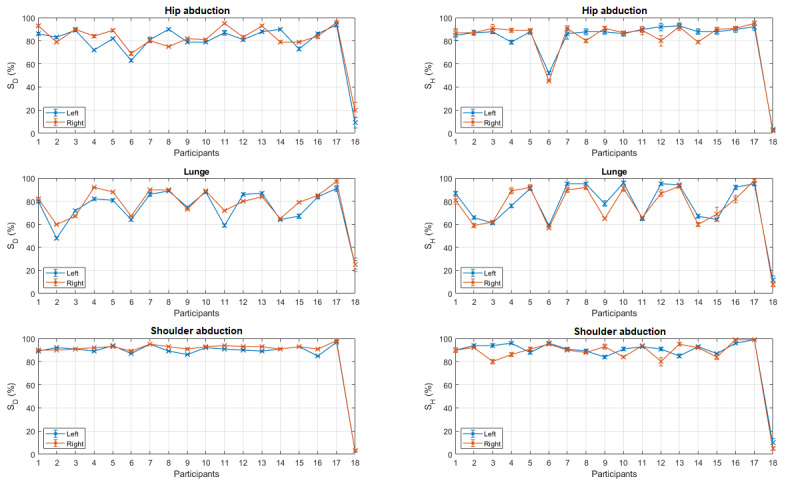
Comparison between left and right scores. Error bars indicate standard error.

A comparison between the left and right performances, excluding sit-to-stand, is shown in Figure 12. No significant differences were observed between the left and the right scores. This was verified by applying a *t* test over the scores for each measure (*P* values are given in Table 1).

Furthermore, a comparison between female and male participants is shown in Multimedia Appendix 1. The scores were averaged across 8 female and 8 male participants (excluding reference). As it can be seen, there was no significant difference between the 2 genders. This was verified by applying a *t* test over S_D_ (*P*=.22) and S_H_ (*P*=.86).

**Table 1:** *P* values obtained by applying the t test over the left and right scores.

Metrices	Exercises		
	Hip abduction	Lunge	Shoulder abduction
S_D_^a^	.27	.09	.44
S_H_^b^	.85	.25	.45

^a^S_D_: dynamic time warping score.

^b^S_H_: hidden Markov model score.

### Conclusions and Future Remarks

We implemented and compared 2 commonly used machine learning algorithms, DTW and HMM, to objectively evaluate the performance of patients using a rehabilitation exergaming platform. 3D movement data were obtained using the Kinect depth camera, and invariant features (joint angles) describing each exercise were extracted. The extracted features are independent of body fit, size, and position and distance of the user to the Kinect. They are also independent of the hardware being used and can be adapted for any motion-sensing device capable of tracking human skeleton joints, such as those mentioned in the *Related Work* section.

Setting a physiotherapist performance as the *reference* and making no movement as the *worst performance*, we applied both DTW and HMM algorithms to compare participants’ performance and report a similarity score. The idea of worst performance was the key to converting the distance measures (obtained from DTW) and likelihood values (obtained from HMM) to similarity scores between 0% and 100%. Overall, both algorithms showed similar trends but had different sensitivities. DTW was observed to be more sensitive to small changes, whereas HMM was more sensitive to the boundaries and clusters resulting from the quantization process. Both DTW and HMM are inherently more sensitive to range of motion than duration. In addition, both measures are sensitive to the worst performance. This suggests ways to use both algorithms to monitor patient progress at different stages: monitoring could start with HMM similarity scores in early stages for a more general comparison and switching to DTW similarity scores in later stages for finer comparison.

The application of these similarity scores is twofold. The scores can be used by the patients at home to encourage them to continue practicing the exergames to achieve higher similarity scores. In addition, the scores can be reported back to the physiotherapist to monitor patient progress and provide feedback. The exercise program can also be adjusted by the physiotherapist given the level of progress to better fit the patient’s needs and progression.

Our proposed method has the potential for significant impact in the context of rehabilitation exergames by enabling remote therapy home-based sessions where performance can still be adequately monitored. This can help better assess the quality of physical exercises performed by patients, fine-tune rehabilitation programs, and enhance the efficiency of home-based rehabilitation. In turn, cost reductions and freeing up of physiotherapy unit time may also be achieved.

Future work will include testing our proposed system on a public data set such as the University of Texas at Dallas-Multimodal Human Action Dataset [44]. In addition, we intend to recruit patients with reduced movement range or other constraints to participate in a study to validate our proposed performance metrics in a clinical setting. The study will also investigate the correlation between the objective scores used by our system and the subjective notes taken by physiotherapists when observing patients performing the exergames.

## Appendix

Multimedia Appendix 1 Comparison between female and male participants, left: DTW scores (SD) and right: HMM scores (SH). Error bars indicate standard error.

## Abbreviations

3D: 3-dimensional
DTW: dynamic time warping
HMM: hidden Markov model
KEHR: Kinect-enabled home-based rehabilitation system
MIRA: Medical Interactive Recovery Assistant
