# Supplementary figures and images for: Rehabilitation Exergames: Use of Motion Sensing and Machine Learning to Quantify Exercise Performance in Healthy Volunteers
Source: JMIR Rehabil Assist Technol. 2020 Aug 18;7(2):e17289. doi: 10.2196/17289 (PMC7463392; doi:10.2196/17289)

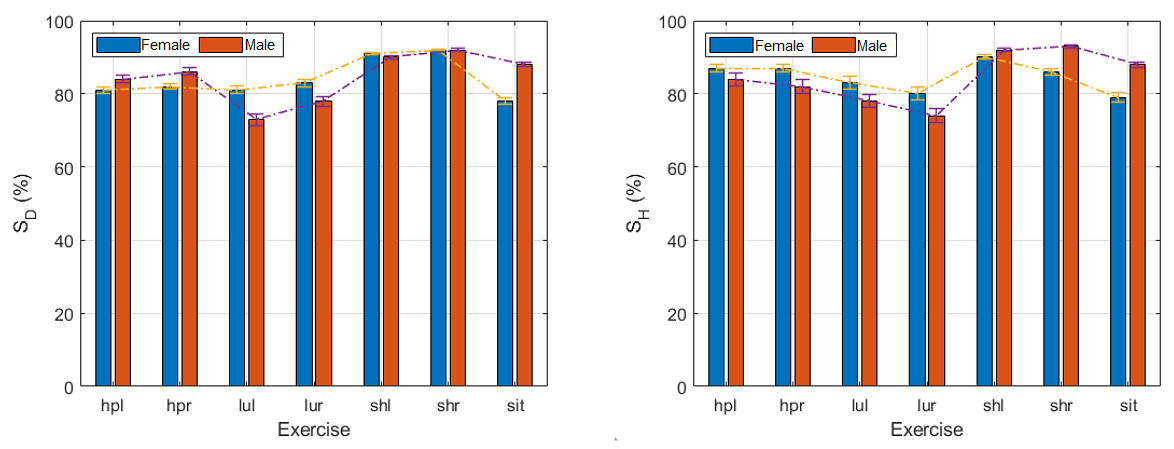

Supplement: Multimedia Appendix 1 [file rehab_v7i2e17289_app1.png]
